# Network-based Responses to the Psychomotor Vigilance Task during Lapses in Adolescents after Short and Extended Sleep

**DOI:** 10.1038/s41598-019-50180-6

**Published:** 2019-09-26

**Authors:** M. W. DiFrancesco, T. Van Dyk, M. Altaye, S. P. A. Drummond, D. W. Beebe

**Affiliations:** 1 Pediatric Neuroimaging Research Consortium, Department of Radiology, Cincinnati Children’s Hospital Medical Center, Cincinnati, OH USA; 2 College of Medicine, University of Cincinnati, Cincinnati, OH USA; 3 Division of Behavioral Medicine and Clinical Psychology, Cincinnati Children’s Hospital Medical Center, Cincinnati, OH USA; 4 Division of Biostatistics and Epidemiology, Cincinnati Children’s Hospital Medical Center, Cincinnati, OH USA; 5 Monash Institute for Cognitive and Clinical Neuroscience, School of Psychological Sciences, Monash University, Melbourne, VIC Australia

**Keywords:** Sleep, Sleep deprivation, Neural circuits, Human behaviour

## Abstract

Neuroimaging studies of the Psychomotor Vigilance Task (PVT) have revealed brain regions involved in attention lapses in sleep-deprived and well-rested adults. Those studies have focused on individual brain regions, rather than integrated brain networks, and have overlooked adolescence, a period of ongoing brain development and endemic short sleep. This study used functional MRI (fMRI) and a contemporary analytic approach to assess time-resolved peri-stimulus response of key brain networks when adolescents complete the PVT, and test for differences across attentive versus inattentive periods and after short sleep versus well-rested states. Healthy 14–17-year-olds underwent a within-subjects randomized protocol including 5-night spans of extended versus short sleep. PVT was performed during fMRI the morning after each sleep condition. Event-related independent component analysis (eICA) identified coactivating functional networks and corresponding time courses. Analysis of salient time course characteristics tested the effects of sleep condition, lapses, and their interaction. Seven eICA networks were identified supporting attention, executive control, motor, visual, and default-mode functions. Attention lapses, after either sleep manipulation, were accompanied by broadly increased response magnitudes post-stimulus and delayed peak responses in some networks. Well-circumscribed networks respond during the PVT in adolescents, with timing and intensity impacted by attentional lapses regardless of experimentally shortened or extended sleep.

## Introduction

Modern society simultaneously demands an ability to sustain attention (e.g., while learning or driving) and challenges the attainment of adequate sleep^1^. Numerous studies in adults confirm that sleep deprivation negatively impacts cognition, especially sustained attention^2^. Effects of inadequate sleep have been investigated far less in children and adolescents, though there is evidence of increased inattention that can affect behavior, learning and brain development^3,4^. Adolescents may be particularly at risk, with the large majority getting chronically less sleep than is recommended on school nights^5,6^, and evidence that shortened sleep affects attention, classroom learning, and safety on the road^4,7–10^. In addition, brain development continues through adolescence, especially in frontal and parietal regions that underlie attention and other cognitive domains^11,12^. Despite these circumstances special to the adolescent years, we know little about how chronically short sleep impacts adolescent brain function.

The psychomotor vigilance task (PVT)^13^ has been employed for the last 30 years as a sensitive test of sustained attention. This simple measure of reaction time (RT) to repetitive stimuli has become recognized as a highly effective tool for measuring degradation of sustained attention performance under sleep deprivation. Adult experiments have shown that sleep deprivation results in increased average reaction times, errors of commission, and frequency of especially long reaction times (>500 ms) known as lapses^14^. A smaller literature confirms similar effects in adolescents^15,16^.

Functional neuroimaging studies of PVT performance are of considerable interest in sleep research as they reveal brain regions that mediate normal and compromised sustained attention. Knowledge of these regions and their interplay as networks may aid the detection^17^ or mitigation^18^ of the effects of poor sleep. Neuroimaging studies have generally identified regions that become more active when tasks demand attention. These “task-positive” regions include fronto-parietal and sensorimotor areas^19–23^. Conversely, when attending to a task, other regions tend to become less active. These “task negative” or “default-mode” regions include medial frontal, posterior cingulate, and inferior parietal gyri^20,21,23–25^. This “task-negative” network is thought to subserve internally directed cognition that must be suppressed to pay attention to external stimuli^26^. Total sleep deprivation (TSD) in adults tends to suppress activation in task-positive regions during attention-demanding tasks^27–30^, especially during attention lapses^17,31^. Drummond *et al*.^21^ reported the first examination of neuronal substrates of variations in performance (fast vs. slow RT) on the PVT in the context of well-rested and sleep-deprived adults. When comparing slow RT to fast RT, generally interpreted as comparing poor vs. good attention, select task-positive regions were more weakly activated and task-negative regions were more weakly deactivated for slow RT. Sleep deprivation amplified these differences between slow RT and fast RT in task-negative regions, but resulted in greater slow RT responses in task-positive regions, possibly related a compensatory recovery of attention after a slow RT.

To date, studies of how sleep might affect the neural substrates of attention have used a clustered-voxel approach that looks for activation patterns region-by-region. It is now well established that cognitive function is more richly attributed to *networks* of regions that are highly interconnected^32^, rather than discretely functioning regions. A given region, for instance, can participate in multiple distinct circuits^33^. Further, some neurodegenerative conditions may be better understood as disruptions of network integration^34,35^. Similarly, taking a network-based approach could inform our understanding of vigilance lapses both in healthy and sleep-deprived populations, as well as those with frank sleep disorders.

The PVT is characterized by extended periods of vigilance and anticipation between brief response-evoking stimuli. A temporally-resolved assessment of not only evoked network response, but also pre-stimulus activity, could provide insight to explain performance and the impact of sleep disruption.

For the current study, we administered the PVT to a large sample of adolescents after 5-night spans of experimentally-manipulated sleep, contrasting 6.5 hours allowed time in bed per night against 10 hours in bed per night. This allowed us to test the impact of a modest but realistic level of sleep restriction on PVT performance. A subset of adolescents completed the PVT while undergoing event-related fMRI, allowing us to apply a contemporary analytic approach to identify and characterize vigilance-related network activity under the two sleep manipulation conditions. This approach, initially introduced to study brain activity before and after EEG-detected epileptic spikes, afforded exploration of stimulus-related activity both just before (anticipatory) and just after (responsive) PVT stimuli. In addition, the analysis method allowed us to test whether PVT-related networks respond differently (a) during lapses compared to responses with RT in the normal range and (b) after short sleep (similar to what many adolescents experience on school nights^5,6^) versus sleep extension. We anticipated time-resolved network-wide changes in PVT pre-stimulus and post-stimulus activity between lapses and normal RT, with modulation by sleep condition. Although the focus of the current study was on adolescents (as an understudied population at particularly high risk for inadequate sleep), we expected such network changes to expand regional outcomes previously observed in adults.

## Results

### PVT performance

General linear models assessed for differences in median RT and number of lapses occurring during the PVT within-subjects across sleep conditions (HS vs. SS) and between-subjects across administration setting (fMRI vs. non-fMRI), as well as sleep x setting interactions. Table 1 details the response times and lapse counts by sleep condition and setting. There was a main effect for sleep condition; compared to HS, during SS median RT was significantly slower (*F*(79) = 4.3, *p* = 0.040) and there were significantly greater lapses (*F*(79) = 7.1, *p* = 0.009). There was also a main effect of setting; compared to those who completed the PVT outside of the scanner, those who took it during fMRI were significantly slower (*F*(1) = 5.7, *p* = 0.019) and had non-significantly greater lapses (*F*(1) = 1.6, *p* = 0.212). As seen in Table 1, the effect of condition was generally modest (small to medium), but consistently in the same direction across subsamples; there were no sleep × setting interactions (*p* > 0.40), suggesting similar adverse effects of short sleep on PVT performance across settings.

**Table 1:** PVT Performance Across Settings and Sleep Conditions.

	Median RT (ms)	Lapses
**Whole Sample**		
Short Sleep	363.7 + 52.3	13.1 + 13.1
Healthy Sleep	354.3 + 49.4	9.7 + 10.2
Sleep effect*	0.052	0.082
**Non-fMRI Group**		
Short Sleep	355.1 + 47.4	11.9 + 12.1
Healthy Sleep	343.1 + 41.8	8.6 + 7.8
Sleep effect*	0.108	0.114
**fMRI Group**		
Short Sleep	376.8 + 47.4	14.9 + 14.4
Healthy Sleep	371.6 + 55.6	11.4 + 13.1
Sleep effect*	0.018	0.063

Note: Sleep effect expressed as partial eta^2^; conventional benchmarks^68^ for small, medium, and large effects are 0.001, 0.056, and 0.138, respectively.

Exploratory analyses also examined the potential moderating roles of the order in which participants experienced the sleep conditions, as well as their age, sex, and race (dichotomized for analysis as White vs. non-White). Two such analyses were conducted for each potential moderator, one for each PVT outcome. Adopting a Bonferroni-corrected alpha of 0.0125 to account for the multiple exploratory models, no potential moderator showed significant main or interaction effects.

### eICA results

**Figure 1:**
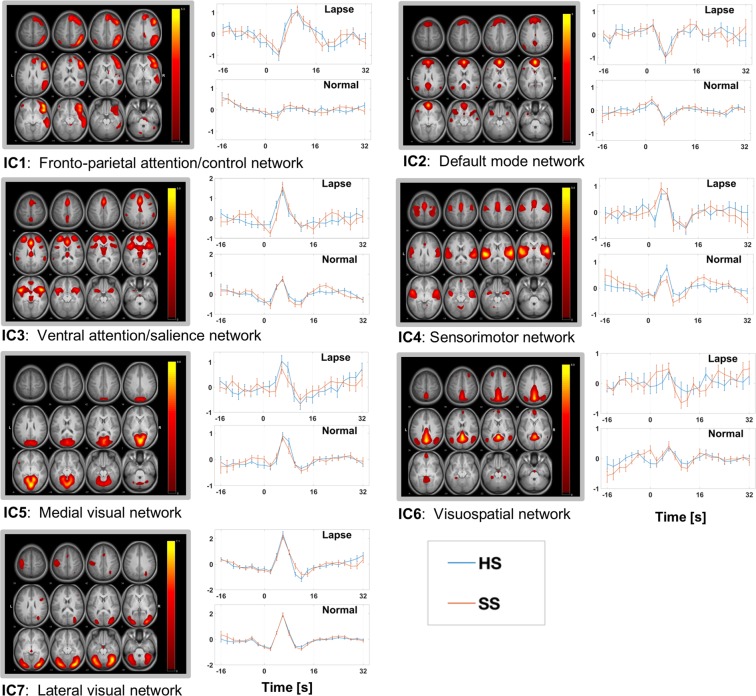
Seven mean eICA group components chosen as relevant to PVT with consistent mean temporal response. Component maps are thresholded at z > 1.5. Time courses are split according to sleep condition (SS (red) and HS (blue)) and RT class (Lapse and Normal, as labeled). Error bars represent standard error. Time t = 0 s corresponds to the PVT stimulus event.

Of the 20 original eICA group components, 7 were retained for further analysis because they showed relatively consistent temporal behavior among subjects and reflected circumscribed networks, in whole or in part, commonly seen in other studies^36–39^: a fronto-parietal attention/control network, default mode network, ventral attention/salience network, sensorimotor network, medial visual network, visuospatial network, and a lateral visual network. The spatial distributions and corresponding mean time courses, separated by sleep condition and RT class, of retained components are shown in Fig. 1. Anatomical brain regions comprising each component network are provided in Table 2. Three additional group components plausibly represented networks of interest, but had inconsistent time courses by our eICA processing. These components are included for reference in Supplementary Fig. S1. The remaining components were excluded because their spatial patterns included the brain periphery or ventricles suggesting the influence of artifacts (e.g., motion artifact that was subthreshold for image exclusion).

**Table 2:** Brain regions comprising component networks.

Component Network	Brain regions included
IC1: fronto-parietal attention/control	mPFC, dlPFC, IPL, TPJ, midTL
IC2: default mode	mPFC, PCC, angular, aIns, TP, IFG
IC3: ventral attention/salience	ACC, aIns, dlPFC, precuneus, IFG, TPJ
IC4: sensorimotor	Pre/post central gyri, SMA, sTL
IC5: medial visual	Calcarine and lingual gyri, cuneus, fusiform
IC6: visuospatial	Precuneus, cuneus, TPJ, dlPFC
IC7: lateral visual	iOcc, midOcc, midTL, post central gyri

mPFC = medial prefrontal cortex, dlPFC = dorsolateral prefrontal cortex, IPL = inferior parietal lobule, TPJ = tempo-parietal junction, midTL = middle temporal lobe, PCC = posterior cingulate cortex, aIns = anterior insula, TP = temporal pole, IFG = inferior frontal gyrus, SMA = sensory-motor area, sTL = superior temporal lobe, iOcc = inferior occipital lobe, midOCC = middle occipital lobe.

### Time course analyses

Statistical outcomes for the main effects of sleep condition and RT class and their interaction for each parameter for each of the 7 chosen networks were evaluated for significance at an adjusted threshold of p < 0.005 to account for assessment of coefficients in multiple components. Outcomes at 0.005 < p < 0.05 were interpreted as trending significance. Table 3 lists the outcomes for the post-stimulus time window. Pre-stimulus, only IC4, the sensorimotor network, showed trending significance of the effect of RT class for slope (p = 0.008) and intercept (p = 0.02).

**Table 3:** Bootstrap ANOVA coefficient p-values for peak height and latency during the post-event period.

Component Network	Peak Fit Parameter	Sleep Effect	RT Class Effect	Interaction
IC1: fronto-parietal attention/control	height	ns	0.00002**	ns
	latency	ns	ns	ns
IC2: default mode	height	ns	0.00001***	ns
	latency	ns	ns	ns
IC3: ventral attention/salience	height	ns	0.000009***	ns
	latency	ns	0.001*	ns
IC4: sensorimotor	height	ns	0.000007***	(0.008)
	latency	ns	ns	ns
IC5: medial visual	height	ns	0.0006*	ns
	latency	ns	(0.04)	(0.02)
IC6: visuospatial	height	ns	0.0009*	ns
	latency	ns	ns	ns
IC7: lateral visual	height	ns	0.0001**	ns
	latency	ns	ns	(0.03)

ns = not significant; parentheses = trending; *p < 0.005; **p < 0.0005; ***p < 0.00005.

### Outcomes by component

#### IC1: Fronto-parietal attention/control network

In the seconds prior to a stimulus, IC1 became steadily less active on average, regardless of sleep condition or whether the subsequent response was a lapse (see Fig. 1). Parametric analysis found no significant effects of RT class or sleep condition, nor any interaction for slope or intercept. Following the stimulus, IC1 activated in a waveform resembling the canonical hemodynamic curve. Mean waveforms shown in Fig. 1, however, revealed greater amplitude and an apparently delayed peak for lapses compared to normal RT. For lapses, parametric analysis found a significantly greater amplitude of response in IC1, though failed to detect a difference in latency (Table 3). These outcomes suggest a more vigorous network response during a lapse. There was no significant effect of sleep condition, nor a significant interaction of sleep condition with RT class.

#### IC2: Default mode network

There is a slight increase in mean activity for IC2 leading up to the stimulus event, regardless of sleep condition or RT class (see Fig. 1). This contrasts with the fronto-parietal pre-event behavior in IC1 and may suggest a gradual strengthening of default mode engagement with increasing time since the previous stimulus. Time course analysis did not, however, find any significant sleep or RT class effects in this time window. Temporal response is negative upon presentation of stimulus, with a significantly stronger peak deactivation for lapses compared to normal RT, complementing the robust positive response for IC1. Lapses were not accompanied by a significantly different deactivation latency. There was no significant main or interaction effect involving the sleep condition.

#### IC3: Ventral attention/Salience network

Mean time courses for IC3 showed a trend towards different pre-stimulus activation patterns, with the HS condition showing a downward trend like the attention network, but a discernible break in this behavior during SS. This was similar for the time periods preceding both lapses and normal RT. Post-stimulus, this network’s activation resembles the response of the attention/control network with a well-defined peak response, again more robust for lapses than normal RT response on within-subject analysis. Peak activation in the ventral attention/salience network was also significantly later for lapses compared to normal RT. No other significant effects were found post-event.

#### IC4: Sensorimotor network

Prior to the event, there was limited mean motor network activity for lapses, but decreasing activity prior to normal responses for IC4. This was reflected by trending differences in slope and intercept between RT classes parametrically. Neither slope nor intercept differed across sleep conditions. In general, there was a significantly more robust post-stimulus peak response after a lapse than normal RT, but there was a trend towards moderation of this effect by sleep condition. Normal RT showed a particularly less robust response during SS. One interpretation of this is that short sleep may weaken motor network response for routine reactions, but there is a compensatory increase in response strength during lapses to match the HS response. There was no significant main effect of sleep condition on post-stimulus peak amplitude and no significant sleep, RT class, or interaction effects on peak latency post-stimulus.

#### IC5: Medial visual network

Prior to the stimulus, activation remained steady in IC5, which fits with the consistent presentation of the neutral blue box. Post-stimulus, peak activation was stronger for lapses than normal RT. There was also a trend towards a difference in latency, with slightly slower peak activations for lapses during SS than HS, and no such differences for normal RT. Again, there was no main effect of sleep on either peak activation latency or amplitude.

#### IC6: Visuospatial network

Pre-stimulus, mean timecourses for IC6 suggest a steeper rising slope for SS compared to HS for normal RT. Interestingly, this may indicate that under short sleep, the regions of this network may be more deactivated but rising on the approach to stimulus events. Parametric analysis, however, did not capture a main effect of sleep on pre-stimulus slope. The stronger effects for this IC were post-stimulus, when lapses were accompanied by peak activations that were significantly stronger after lapses. There was again no significant effect of sleep condition on post-stimulus response.

#### IC7: Lateral visual network

Relative to other networks presented here, the mean response of this network is remarkably stable across subjects for either RT class or sleep condition. This low variability aided discernment of a RT class effect for peak height in the bootstrapped ANOVA with, again, higher peaks during lapses compared to normal RT responses. An interaction effect, between sleep condition and RT class, of trending significance was also observed for peak latency for this network. No other effects pre-event or post-event were found.

## Discussion

This is the first study of response to a vigilance test in adolescents exposed to chronic sleep restriction relevant to everyday experience for this population. Consistent with prior adult studies and with our previously-reported behavioral outcomes^10,40^, five nights of modestly shortened sleep – similar to that experienced by many adolescents regularly on school nights – significantly diminished vigilance on a computerized measure. These sleep-related effects were similar whether subjects were sitting upright outside the MRI scanner or lying down in an MRI scanner. We capitalized on the fact that the sleep manipulation had a similar effect within the scanner as outside of it to probe brain responses to the PVT in a novel fashion. The approach was to apply the PVT during fMRI to a group of healthy adolescents (a) to clarify, in a network-focused manner, how the brain responds to this task, which has proven to be the most sensitive to sleepiness-related vigilance deficits and (b) to probe network-wide differences both pre- and post-stimulus in activity across response lapses versus normal RT, and across chronic short sleep versus a well-rested state. We capitalized on a recently-described analytic approach, eICA, to identify 7 eICA components with consistent time courses representing distinct functional networks involved in sensory processing, attention, control, vigilance to stimuli, and motor function for the PVT task. Additionally, our analytical approach of parametrically modeling the time courses effectively interpolated the response between time points separated by imaging repetition, allowing fine-scale comparison of magnitude and evolution of temporal response of each network by sleep condition and by RT class (normal RT vs. lapses).

The networks delineated by eICA for the PVT are well recognized as canonical resting-state networks and are comprised of regions well-associated^41^ with maintenance of attention and executive control. They can be separated as “task-positive” networks stimulated by repeated targets/responses and a “task-negative” network, often called the “default mode,” that disengages to attend to the targets. That “default mode” network, which anteriorly involves medial frontal regions and posteriorly involves the cingulate and angular gyri, is known to be more active during daydreaming and calm introspection, which presumably must be suppressed when actively attending to and responding to an external stimulus. The spatial pattern of IC2, generated by eICA, matches well the recognized task-negative default mode network^37,39,42^. As expected, IC2 deactivates post-stimulus as task-positive networks, such as the attention/control network (IC1) are engaged.

The remaining networks, all of which activate upon stimulus presentation, serve distinct roles for stimulus perception, sustained attention, and response generation.

In particular, the fronto-parietal attention/control network (IC1) and the salience network (IC3) are key for alerting, top-down short-term attention processing, and maintaining vigilance to salient external stimuli. Previous studies^19,23,43–45^ have described brain regions comprising lateral frontal areas and portions of the inferior parietal lobule as together supporting attention and executive control. Attention processing is lateralized in the brain, depending on the type of attentional challenge^20,46,47^. Sustained attention tasks like the PVT predominantly activate a task-positive right-sided fronto-parietal network as represented by IC1. The eICA also generated a left-lateralized homologue to IC1 (shown in Supplementary Fig. S1) but it lacked a peaked mean response to stimuli, suggesting a potentially less stimulus-dependent role for the left-sided network. The component IC3 is well recognized as the salience network, including the anterior insula, anterior cingulate, and lateral prefrontal regions as major components, that is involved in the detection of prominent or important target inputs, such as the counter in the PVT^45,48,49^. The salience network is responsible for alerting and for moderating the switch between the default mode and the attention/control networks^49^.

Sensorimotor processing of the button press is supported by pre/post central regions, as reflected in IC4. Salient sensory input for the PVT used in this study is entirely visual and the remaining three networks support various levels of visual processing. The regions in IC5 comprise the extrastriate visual cortices; the lingual, calcarine, and fusiform gyri. In the context of the PVT, this network likely serves target processing as part of visual attention^19^; reacting to the appearance of the counter in the blue box. The visuospatial network, IC6, plays a key role in vigilance to visual input. The precuneus and posterior cingulate represented in IC6 can be associated with the default mode network, but these regions are also implicated in a “sentinel” role^50,51^, attending to expected repeated stimuli as experienced in the PVT. The IC7 network well describes the ventral visual stream^52^, including primary visual cortex, fusiform gyri and regions of the inferior temporal lobe. It subserves basic visual processing and object or shape recognition, the most primitive input processing of PVT stimuli.

A common outcome for all networks, regardless of sleep condition, was that lapses evoked a stronger peak response post-stimulus compared to normal RT. Prior experiments with the PVT in adults under event-related fMRI have compared the slowest 10% of RT to the fastest 10% of RT^21,31^. Drummond *et al*.^21^, using a PVT task similar to ours, found clusters in the basal ganglia, fronto-parietal cortices, and motor regions where activation to slow RT decreased compared to fast RT. This prior work also found that the medial prefrontal cortex, comprising the anterior portion of the default mode network, exhibited weaker deactivation in response to slow RT than fast RT. If using slow RT as a surrogate for lapses, these results are in conflict with ours. In contrast, Chee *et al*.^31^, using a selective attention task, reported increased peak signal in frontoparietal regions for slower responses compared to faster responses. They explain the augmented signal as a consequence of diminished processing efficiency. The increased network responses to lapses observed in this study align with Chee *et al*.^31^ and may similarly be explained by lowered network processing efficiency, despite the fact that our task better resembles the one used by Drummond *et al*.^21^ Methodologic differences may explain the contradictions between the three studies. The two prior studies imposed sleep deprivation in contrast with the chronic sleep restriction protocol used in this study. Furthermore, designation of fast and slow response extremes was made relative to each subject in the previous studies, whereas our study relied on an absolute threshold for lapses and enveloped the fastest responses in each individual into the designation of normal RT. In addition, we have done network-wide assessments of response that may not reflect results in individual regions. Finally, our population is solely comprised of adolescents whose brains may exercise different strategies for handling lapses or slower responses compared to adults. Future studies could explore prospectively distinctions between adolescent and adult processing during lapses.

Only one prior study sought specifically to differentiate pre-stimulus from post-stimulus vigilance activity^24^. Our analysis approach, by design, defines temporal periods pre- and post-stimulus *a priori*, and uses curve-fitting approaches to allow for empirical interpolation between the relatively sluggish fMRI data collection points. In doing so, this study demonstrated a slow deactivation of the fronto-parietal attention/control network and complementary rise in activation of the default mode network prior to stimulus onset. This may reflect a natural tendency towards drifts in attention over time. However, we cannot rule out the potential influence of methodological artifact: the random inter-stimulus interval of the PVT means that the pre-stimulus time period can reflect “carryover” from earlier stimuli that, because of their random timing, could create greater “noise” the further away one gets from a target stimulus. Importantly, aside from statistically significant and trending outcomes for IC4 that may be worthy of follow-up in future studies, network activity pre-stimulus was not systematically related to sleep condition, the presence of a subsequent lapse, or the interaction of the two.

In general, quantitative imaging outcomes did not exhibit a strong effect of sleep condition. Qualitative analyses of Fig. 1 suggest some delay in executive control (IC1) and weakening of visual target detection response (IC5) for lapses after experimentally shortened sleep, but quantitative comparisons resulted in no main sleep effects and only trends for interaction between sleep condition and RT class effects in the visual domain (IC5 and IC7) and in the sensorimotor domain (IC4). One prior study using a shorter but more extreme sleep deprivation protocol in adults performing the PVT also found no main effects of sleep condition, and limited sleep-by-RT interactions^21^. However, Chee *et al*.^31^, employing a selective attention task, describe weaker peak activation associated with lapses when sleep deprived compared to a well-rested state. The reduced response was attributed to dampened recruitment of frontoparietal neurons to compensate for lapsing in the sleep deprived state. Although it is possible that these discrepancies in findings related to task differences (sustained vigilance vs. selective attention), this is only one of several methodological differences across studies, so further work is needed. Based upon the commonalities across the two PVT studies, we tentatively suggest the higher-level aspects of daydreaming may be the same when sleep restricted as when well-rested; but daydreaming episodes may simply be more common after sleep restriction due to less activity of lower-level arousal mechanisms. It is worth noting that those lower-level arousal mechanisms are mediated via mesencephalic and diencephalic regions that are not intensively probed via telencephalic-oriented fMRI protocols such as that used here.

Alternatively, our protocol may not afford sufficient signal for imaging to detect. Our lengthened version of the PVT (around 1/3 longer than is typical) was intended in part to increase sensitivity to inadequate sleep (increasing the opportunity for lapses), but that may not have resulted in a stronger signal on a response-by-response basis. We are not aware of other published studies on the PVT in adolescents tested lying prone in a scanner, but our tabletop findings show reaction times in well-rested adolescents were similar to what others have reported^15^. While our sleep restriction protocol was intentionally chosen to study common adolescent sleep behavior, a greater effect may be evident after more marked sleep restriction, as has been reported by others who have used more extreme curtailment of sleep^15,16,53^. An incidental finding from this study was that reaction times were slower overall for adolescents assessed in the MRI setting versus during a tabletop administration, despite similar demographics and adherence to the sleep manipulation. Carr *et al*. reported a similar effect of the MRI environment on PVT performance^54^ (i.e., worse performance in the scanner than outside of it). Despite limited findings of effects of sleep condition, the results of this study will aid design of future studies that investigate this apparent “scanner effect”, and that employ more extreme (albeit less ecologically-relevant) sleep restriction, larger cohorts, or both, to ascertain better the impact of sleep restriction on networks supporting sustained attention under the PVT.

Our conjecture that temporal drifts in attention may influence task-positive and task-negative network responses suggests a potential dependence of our measures on ISI. It has been reported that short ISI (<5 seconds) in the PVT introduce greater variance of RT, with shorter ISI resulting in slower RT^55^. This tendency may be due to a lack of preparation afforded by very short ISI. In accordance, we observed a significant increase in 1/RT as a function of ISI for our PVT (see supplementary Fig. S2). Since our PVT included a wide range of ISI, including intervals shorter than 5 seconds, we followed the advice of Matthews *et al*. and explored the impact of excluding this range of ISI on our eICA outcomes. With responses for 3 and 4-second ISI relegated to covariates in the FIR stage of processing, eICA resulted in the same networks with similar parametric outcomes (see supplementary Fig. S3 and Tables S1 and S2). Some notable exceptions include an apparent lag in SS default mode (IC2) mean response compared to HS for lapses (Fig. S3) and detection of parametric sleep effects during the pre-event period for the visual component IC5 (Table S1).

This study has limitations that warrant discussion. As already noted, the modest sample and realistic but mild sleep restriction protocol may have limited statistical power of final analyses. The variability of deconvolutions comprising the eICA may also have been impacted by the relatively limited number of lapse events (averaging approximately 11 to 15, see Table 1) compared to events with normal response. However, prior application of eICA to absence epilepsy relied on as few as 5 seizure events for deconvolution^56^. Also, there is an inherent trade-off between temporal and spatial data acquisition in fMRI. As with prior fMRI studies of the PVT, we sought to cover the full neocortex, but this resulted in 2 second intervals between data acquisition points that necessitated statistical interpolation via curve-fitting. Future studies with more spatially-limited foci and/or faster multi-band image acquisition techniques could offer greater temporal resolution, as could studies using other technologies that are highly temporally sensitive but offer less certain spatial resolution (e.g., magnetoencephalography). Complementary studies using other technologies could also bypass the potential that the observed hemodynamic responses (upon which fMRI is based) could be broadly dependent on vascular changes due to sleep-related factors^57,58^. Reassuringly, we observe our strongest within-subject effects for lapses regardless of sleep condition. Finally, our sample was comprised exclusively of healthy adolescents. While this choice was motivated by a desire to better understand the impact of sleep restriction in a group highly exposed to short sleep and undergoing rapid brain development, it remains to be seen how well present findings generalize to other developmental stages or to adolescents with clinical sleep disorders.

Despite these limitations, this study was the first to apply network-based analyses to understanding brain activation in the context of the most widely-used measure of cognition in the sleep literature. Further, it was one of the first to probe for changes in brain activation in response to sleep restriction in any pediatric sample, focusing on the particularly high-risk developmental period of adolescence. In doing so, it clarified the brain networks involved in the PVT, shed new light on differences in network activity that may underlie attention lapses, and helped to define design limits on future studies seeking to understand the impact of chronic sleep restriction on cognitive performance.

## Methods

The study protocol was approved and overseen by the Institutional Review Board at Cincinnati Children’s Hospital Medical Center. All methods were performed in accordance with relevant guidelines and regulations. Informed assent was obtained from all participants and written informed consent was given by all parents and/or legal guardians.

### Subjects

This fMRI study included 14.0–16.9-year-old healthy adolescents who were drawn from a larger study of the impact of chronic sleep restriction on adolescent functioning^40,59^. These adolescents were selected at random from those who successfully completed the PVT and other computerized attention tasks during an initial study visit, prior to any sleep manipulation. A total of 50 were selected to undergo fMRI while completing the PVT during two separate study visits. An additional 71 completed the identical task outside of the fMRI environment, while seated upright at a table. All were free of known neurological or psychiatric disorders or contraindications for MRI. Full recruitment, inclusion, and exclusion criteria are detailed in prior papers^10,40,59^. Of those selected for fMRI, 18 were excluded from analyses (9 were found to have excessive imaging artifacts during at least one session due to motion; 5 subjects did not complete MRI acquisition due to discomfort, trouble fitting in the head coil, or because they failed to return for a second scan; 3 were found to be non-adherent to the sleep protocol or PVT task (as defined below); 1 was missing PVT data due to a technical failure). Of those selected to complete the PVT outside of the scanner, 22 were excluded from analyses due to non-adherence to the sleep protocol or PVT. See Table 4 for demographic and sleep characteristics for the final sample of 32 adolescents who underwent fMRI and 49 who completed the PVT outside of the scanner, as well as for those excluded from analyses.

**Table 4:** Demographics and objective sleep parameters for the final fMRI and non-fMRI samples and for those excluded from analyses.

	Excluded Sample (*N* = 38)	Final fMRI Sample (*N* = 32)	Final non-fMRI Sample (*N* = 49)
Demographics			
Age	15.5 ± 1.00	15.7 ± 0.75	15.8 ± 1.04
Female Gender	24 (63.2%)	22 (68.8%)	27 (55.1%)
Race			
White	14 (36.8%)	17 (53.1%)	23 (46.9%)
Black	21 (55.3%)	14 (43.8%)	19 (38.8%)
Other	3 (7.9%)	1 (3.1%)	7 (14.3%)
Healthy Sleep Actigraphy Data			
Onset – time**	22:51 ± 1:08	22:03 ± 0:56	22:06 ± 0:42
Offset - time	7:10 ± 0:41	7:08 ± 0:32	7:12 ± 0:33
Sleep Duration – hours***	8.3 ± 1.17	9.1 ± 0.64	9.1 ± 0.64
Short Sleep Actigraphy Data			
Onset – time*	24:56 ± 0:54	24:32 ± 0:40	24:39 ± 0:44
Offset - time	7:31 ± 0:47	7:02 ± 0:29	7:15 ± 0:37
Sleep Duration - hours	6.6 ± 0.82	6.5 ± 0.53	6.6 ± 0.51

*p < 0.05, **p < 0.01 in ANOVA analyses comparing the three groups. Where group effects occurred, the final fMRI and non-fMRI samples did not significantly differ, but those excluded from analyses differed from those included in final analyses.

### Sleep manipulation

Study participants underwent a 3-week within-subject randomized cross-over protocol^60^: an initial sleep-stabilization week followed by 2 experimental weeks, including 5 consecutive nights of short sleep (SS, 6.5 hours in bed) or healthy sleep (HS, 10 hours in bed). Wake time was held constant throughout the study, and set at the time participants would need to rise to come to the office for an 8:30 am visit. The initial week was intended to set and stabilize circadian phase by allowing participants to self-select bedtimes, whereas bedtimes were set to match the in-bed requirements during the SS and HS conditions. SS and HS were each preceded by 2-night wash-outs identical to the initial sleep stabilization condition.

Participants were required to avoid napping and to limit caffeine intake to no more than one coffee or energy drink per day or no more than two caffeinated sodas per day. Daily sleep diaries and actigraphy (MicroMotionlogger Sleep Watch, Ambulatory Monitoring, Inc.) monitored adherence to the sleep protocol. Actigraphy data were uploaded and reviewed with each adolescent and family during morning office visits immediately following the initial phase stabilization, SS, and HS conditions. Trained research associates queried any inconsistencies between the diary and actigraph results, and screened for artifacts (e.g., actigraph removal). Artifact-free actigraphy data were run though a validated algorithm^61^ to determine each adolescent’s average sleep onset, offset, and period (offset minus onset, regardless of awakenings during the night) within each sleep condition. Adolescents who averaged less than 1/2 hour difference in nightly sleep period across the SS and HS conditions were considered non-adherent to the sleep protocol. Because the sleep outcomes were roughly normally distributed, paired-sample t-tests were used to examine cross-condition differences (see Table 4).

### PVT paradigm

Each adolescent completed the PVT between 8:00 and 11:00 in the morning at the end of HS and SS, with each participant being tested at the same time of morning across both sleep conditions. The psychomotor vigilance task (PVT) implemented in the scanner for this study was similar to the one developed by Drummond *et al*.^21^ Presentation® software (Neurobehavioral Systems, Inc.; Albany, CA), running on a dedicated computer, controlled stimulus display and response logging. Those who completed the PVT outside of the scanner sat comfortably upright viewing stimuli on a 16″ diagonal computer monitor at eye level, responding with the space bar on a standard computer keyboard. Those who completed the PVT while lying in the scanner viewed the stimuli via MR-compatible video screen and responded via a handheld button box (Current Designs, Inc.; Philadelphia, PA). Task programming was identical across settings. In the center of the screen appeared a blue rectangle against a black background. At interstimulus intervals (ISI) ranging between 3 and 11 seconds, a millisecond timer appeared in the box and immediately started counting time. Subjects were instructed to press the space bar or button box as soon as possible after the start of the counter. The button press stopped the counter and displayed the resulting reaction time (RT) for 500 ms as immediate performance feedback. Stimuli were presented so that ISI were equally represented among the values of 3, 4, 5, 6, 8, 9, 10, and 11 seconds throughout the task. This was achieved by using all 8 ISI, randomly shuffled, in each of 14 consecutive sets for a total of 112 stimuli shown over a task duration of approximately 13 minutes. In the rare event that time to respond exceeded 2.4 seconds, subjects heard an alarm through headphones and viewed a prompt to respond. Mean RT and number of lapses (defined as RT > 500 ms) were computed for each subject in each condition. Non-adherence to the task was defined as holding the space bar down continuously across numerous stimuli (for non-fMRI subjects) or limited engagement with the tasks, as reflected in > 75 lapses during either assessment session (1 fMRI and 1 non-fMRI subject).

### MRI protocol

All subjects who underwent MR scanning did so on a 3-Tesla Philips Achieva MRI system (Philips Research, Eindhoven, Netherlands) equipped with an 8-channel head coil. Each session included a high-resolution structural image acquired via 3D-MPRAGE^62,63^ with echo/inversion/repetition time (TE/TI/TR) = 2.9/903/6.8 ms, field of view = 176 × 256 × 256 mm, and 1 mm^3^ voxel size. T2*-weighted blood oxygenation level-dependent functional images were acquired continuously during task execution with a gradient-echo echo-planar imaging sequence covering 33 axial slices, 4 mm thick, TR/TE: 2000/30 ms, field of view = 256 mm^2^, and matrix of 64 × 64. The PVT required acquisition of 402 images, with the initial 6 images discarded from analysis to allow for attainment of T1 relaxation equilibrium.

### fMRI preprocessing

Data from the 3D anatomic and fMRI acquisitions were processed under Statistical Parametric Mapping software (SPM8, Wellcome Trust Centre for Neuroimaging, www.fil.ion.ucl.ac.uk/spm) in the Matlab computing environment (The Mathworks, Inc., Natick, MA). The functional images were realigned to the first image in the series. This was followed by co-registration of the anatomic image to the mean realigned functional image. Anatomic images were spatially normalized to Montreal Neurological Institute standard space via transformation parameters resulting from segmentation in SPM8 based on gray matter, white matter, and cerebrospinal fluid templates^64^. The same transformation was applied to the functional image series before Gaussian smoothing at 8 mm full width at half maximum.

Preprocessing concluded by performing quality assurance on the fMRI series with the aid of the automated Artifact Detection Toolbox (http://www.nitrc.org/projects/artifact_detect/). Images in any series undergoing composite motion greater than 3 mm or an intensity change of z-score greater than 3 relative to adjacent images were marked and rejected as a covariate in further analysis. Imaging series were also visually assessed to exclude for artifacts not captured by the automated toolbox.

### eICA method

Preprocessed fMRI data from the PVT were analyzed using event-related independent component analysis (eICA). This method, first developed by Masterton *et al*.^65^ to address the temporal signature of network activation resulting from epileptiform brain activity^56^, is a specific application of independent component analysis (ICA). ICA is a data-driven approach to allow decomposition of spatiotemporal data in such a way that maximizes the statistical independence of resulting spatial components, each with a corresponding weighting of the original data over time^66^. Group ICA performs decomposition of a group of datasets, spatially stacked^67^, resulting in group-level components that can each be subsequently back-projected for representation by individual datasets. This approach separates fMRI-acquired voxels into spatial patterns or networks (components), each with a corresponding network-wide time course covering the period of image series acquisition.

eICA builds upon the ICA approach by linking networks and their corresponding time courses to specific types of events. In lay terms, eICA identifies clusters of brain regions (called “components”) that activate or deactivate similarly relative to known events. We applied eICA to PVT responses to determine the temporal network response both as a result of and in anticipation of PVT stimulus presentation events. For comparison purposes, we separated those events according to sleep condition (SS vs. HS) and two response time classes (lapses = RT > 500 ms; normal responses = RT 200–450 ms). The 50 ms gap in RT range between normal and lapsed responses was imposed to avoid ambiguity when assigning RT class. RT values recorded as < 200 ms were considered spurious and removed from consideration.

The eICA pipeline begins by fitting the preprocessed fMRI series from each session, voxel-wise, to a finite impulse response model to, on average, deconvolve event responses using 24 time points, one every TR (2 seconds) covering the period from 16 seconds prior to each stimulus event to 32 seconds following each event. This choice of temporal coverage was to maintain consistency with Masterton *et al*.^56,65^ and so that we could examine both anticipatory and post-stimulus activity for the PVT. The finite impulse response model was expanded to separately fit events according to RT class. The resulting model thus included 48 parameters (24 for each RT class: lapses and normal responses), with covariates including parameters representing the responses shorter than 200 ms or between 450 and 500 ms, the 6 motion parameters, and any outlier images from the automated artifact detection processing.

The eICA pipeline continues by considering the resulting 48 deconvolution parameter maps for each subject as an image time series for input to group ICA, carried out by the Group ICA of fMRI Toolbox (GIFT v3.0a: http://icatb.sourceforge.net) operating in the Matlab computing environment [Mathworks, Natick, MA]. The Infomax algorithm was utilized, prefaced by two principal component data reduction steps, to generate 20 independent components from a group of 66 datasets (33 subjects, 2 datasets each corresponding to SS and HS). Back-reconstruction then ensued by the GICA approach in GIFT to obtain the representation of each group component per individual dataset. The voxel-wise mean, across datasets, of each back-reconstructed group component was scaled as z-scores and inspected visually after thresholding at z > 1.5. Mean corresponding time courses per group component, including standard error per time point, were also discerned. These mean outcomes were judged for each component as either representing a plausible functional network supporting the PVT with consistent temporal response, or as likely artifact. The back-reconstructed time courses for each subject, separated by RT class (24 points representing normal responses and 24 points representing lapses) and sleep condition, corresponding to the group components chosen as relevant to the PVT, were the focus of subsequent analyses.

### Time course analysis

Beyond simply identifying networks (eICA components) involved in the PVT, this study aimed to examine differences in the timecourse of network activation across the two RT classes (lapse vs. normal) and two sleep conditions (SS vs. HS) for each of three distinct temporal windows (16 seconds pre-stimulus, 0–16 seconds immediately post-stimulus, 17–32 seconds post-stimulus). The choice of 16 s windows was motivated, in part, by the time scale of the canonical hemodynamic response in fMRI. We initially considered the straightforward approach of comparing activation at each of the 24 data collection time points (every 2 seconds). However, this approach was rejected because it was temporally insensitive and either inappropriately treated each data collection point as independent of the others or, in a nested statistical model, failed to account for the presence of common waveforms (e.g., the hemodynamic response). Instead, we derived statistical models within each temporal window (pre-stimulus, immediately post-stimulus, late post-stimulus) that could then, in turn, be parsimoniously summarized by a small number of parameters amenable to comparison across sleep conditions and RT classes.

**Figure 2:**
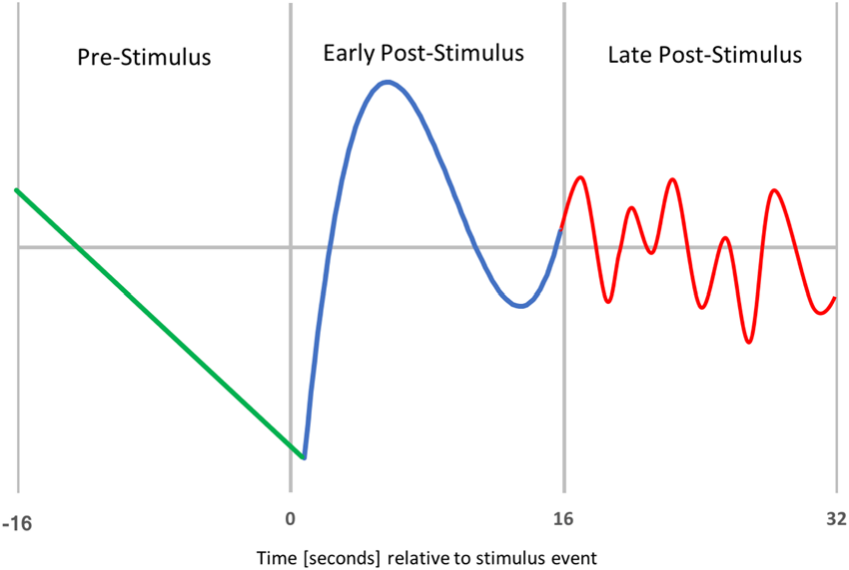
Schematic of modeled response periods. Green: the period prior to the stimulus. Blue: the early post-stimulus period. Red: the late post-stimulus period.

This was accomplished within each temporal window via a least squares fit of each time course to a polynomial of the lowest order such that the parameters captured salient response characteristics. As schematically illustrated in Fig. 2, the pre-stimulus period (−16 seconds to stimulus) was best described, across components, as having a linear signal evolution; the signal tended to rise or fall without consistent “bends.” Given this, the parameters chosen for analyses during the pre-stimulus period were signal slope and intercept for each subject, RT class, sleep condition, and eICA component. For the early post-stimulus period (0–16 seconds post stimulus), the time courses resembled a hemodynamic response in terms of peak timing. The traditional dual-gamma functional representation of the canonical hemodynamic response was considered for modeling post-stimulus response, but it was found to poorly fit the sometimes bi-phasic curves observed. Instead, it was found that its features were best modeled as a third-order polynomial. To efficiently capture salient aspects of this curve, we extracted the height of the first peak and the corresponding latency (time-to-peak) from the stimulus event for each subject, RT class, sleep condition, and eICA component. During the late post-stimulus time window (16–32 seconds post-stimulus), mean responses were either difficult to describe or nearly flat, possibly reflecting a high degree of variability of the contributions from subsequent events. Since this late time window was a challenge to interpret, we chose to limit quantitative analyses to the pre-stimulus and early post-stimulus periods.

**Figure 3:**
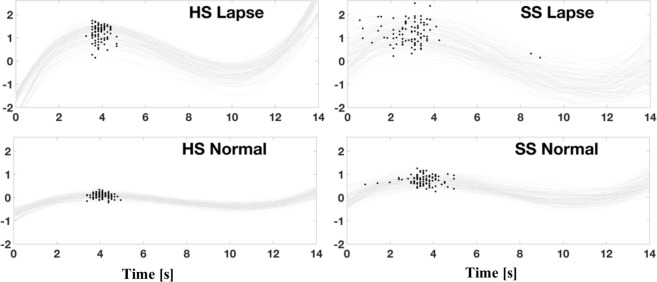
Example bootstrapped resampling of curve fits and peak locations for one subject during the post-event time window of IC6 Visuospatial Component. Gray lines show resampled third order polynomial fits to the group eICA time course for the component. Dots indicate peak locations for the HS and SS curves for lapsing and normal responses, as labeled.

Following the initial fit, variance of the parameters was determined by bootstrapping: the fit was repeated 1000 times, each following a resample of the original residuals with replacement. An example of bootstrap resampling for peak position for one subject during the early post-stimulus time window of one component (IC6, described below) is shown in Fig. 3. Resampled values of a given parameter were then entered into an ANOVA for (RT class) × (sleep condition) for each subject individually. ANOVA coefficients were assessed by a second round of bootstrapping: ANOVA was repeated 1000 times, each following a resample of the fit parameter values in each cell defined by RT class and sleep condition. The mean across subjects of the resulting ANOVA coefficients for the main effect of RT class, main effect of sleep condition, and their interaction were calculated for each eICA component of interest and statistically appraised for significance via single-tail sign-rank test with null hypothesis of zero.

## Supplementary information

Supplementary Materials

## Data Availability

The datasets generated during and/or analyzed during the current study are available from the corresponding author on reasonable request.
