# Supplementary material for: Network-based Responses to the Psychomotor Vigilance Task during Lapses in Adolescents after Short and Extended Sleep
Source: Sci Rep. 2019 Sep 26;9:13913. doi: 10.1038/s41598-019-50180-6 (PMC6763427; doi:10.1038/s41598-019-50180-6)
Supplement: Supplementary file 1 — Supplementary Materials [file 41598_2019_50180_MOESM1_ESM.pdf]

Supplementary Materials

Network-based Responses to the Psychomotor Vigilance Task during Lapses in Adolescents after Short and Extended Sleep

DiFrancesco MW, Van Dyk T, Altaye M, Drummond SPA, Beebe DW

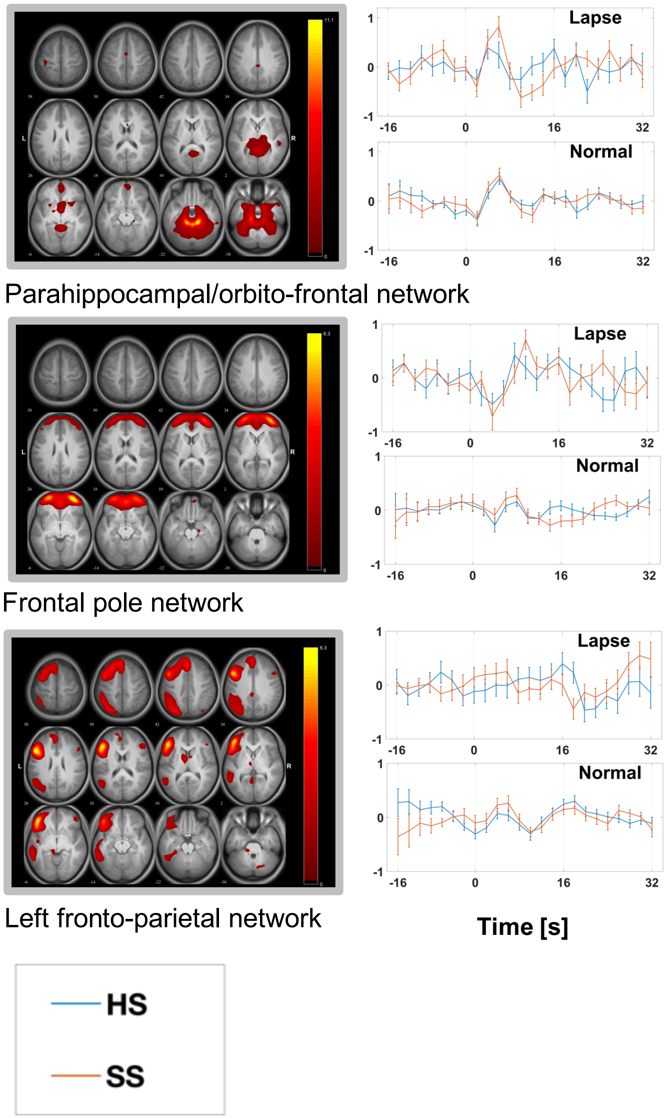

Figure S1: Additional components with recognizable spatial distributions, but with weak or noisy mean time courses. These components were excluded from final analyses.

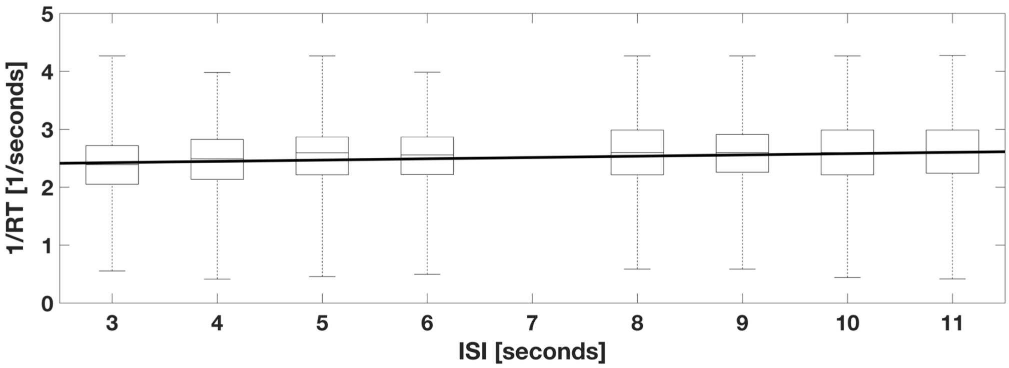

Figure S2: Psychomotor vigilance task (PVT) inverse reponse time (1/RT) vs. interstimulus interval (ISI). The line in each box represents median value and the vertical extents of the boxes represent the 25th and 75th percentiles. Vertical bars represent extreme values.
Fitted slope is significant (0.022 s^{-2}, p = 5 x 10^{-8}).

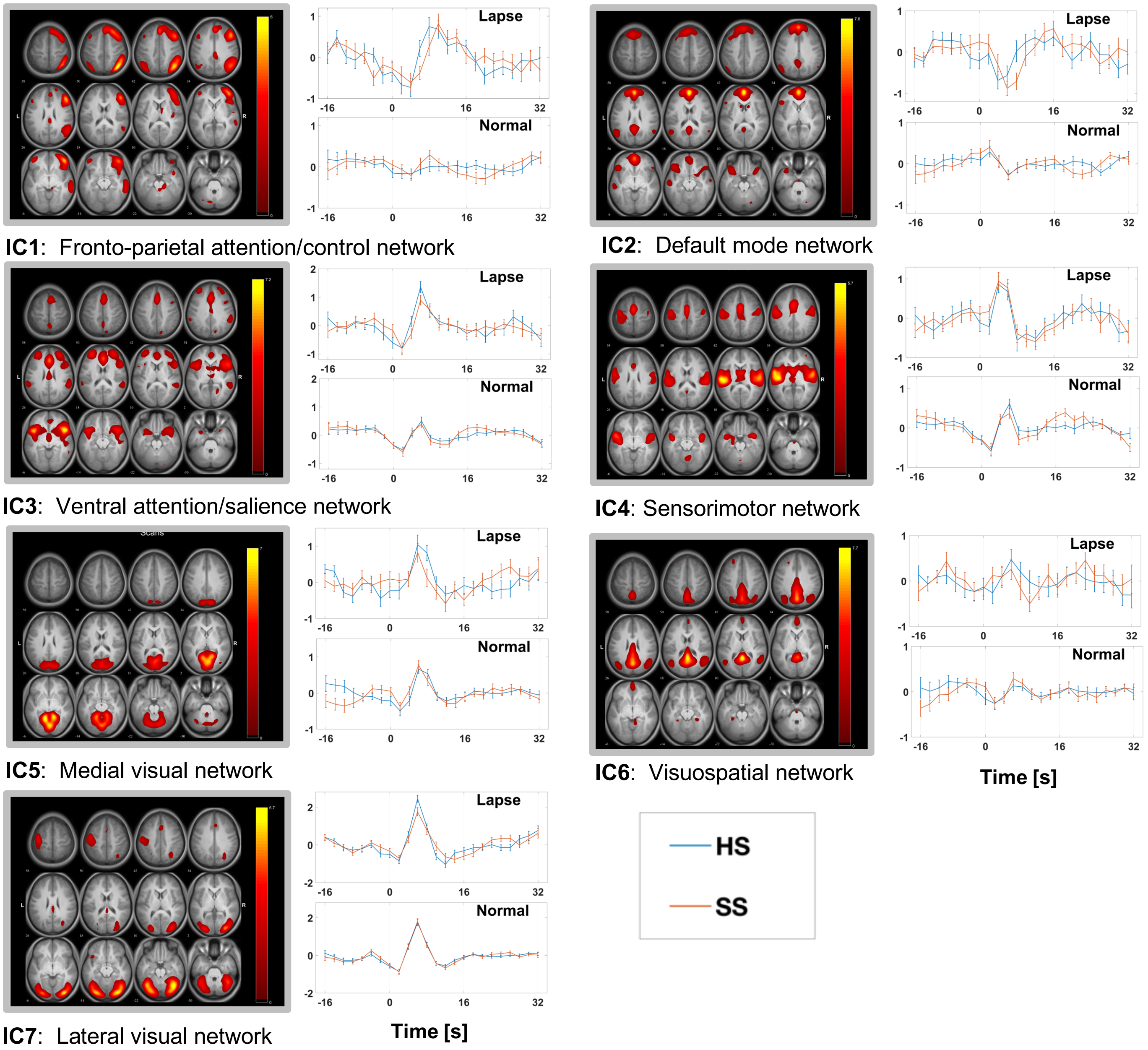

Figure S3: Seven mean eICA group components after exclusion of ISI shorter than 5 seconds. Component maps are thresholded at z > 1.5. Time courses are split according to sleep condition (SS (red) and HS (blue)) and RT class (Lapse and Normal, as labeled). Error bars represent standard error. Time t = 0 s corresponds to the PVT stimulus event.

Table S1: Bootstrap ANOVA coefficient p-values for slope and intercept at the event time during the pre-event period after excluding ISI shorter than 5 seconds.

Component Network	Peak Fit Parameter	Sleep Effect	RT Class Effect	Interaction
IC1: fronto-parietal attention/control	slope	ns	(0.05)	ns
	intercept	ns	0.003*	ns
IC2: default mode	slope	ns	ns	ns
	intercept	ns	ns	ns
IC3: ventral attention/salience	slope	ns	ns	ns
	intercept	ns	ns	ns
IC4: sensorimotor	slope	ns	0.003*	ns
	intercept	ns	(0.01)	ns
IC5: medial visual	slope	0.002*	ns	ns
	intercept	0.003*	ns	ns
IC6: visuospatial	slope	ns	ns	ns
	intercept	ns	ns	ns
IC7: lateral visual	slope	ns	(0.04)	ns
	intercept	(0.03)	ns	ns

ns = not significant; parentheses = trending significance; *p < 0.005

Table S2: Bootstrap ANOVA coefficient p-values for peak height and latency during the post-event period after excluding ISI shorter than 5 seconds.

Component Network	Peak Fit Parameter	Sleep Effect	RT Class Effect	Interaction
IC1: fronto-parietal attention/control	height	ns	0.0001**	ns
	latency	ns	(0.01)	(0.05)
IC2: default mode	height	ns	0.0002**	ns
	latency	ns	ns	ns
IC3: ventral attention/salience	height	ns	0.0001**	ns
	latency	ns	ns	ns
IC4: sensorimotor	height	ns	0.0007*	(0.03)
	latency	ns	ns	ns
IC5: medial visual	height	ns	0.0006*	ns
	latency	ns	ns	ns
IC6: visuospatial	height	ns	0.00006**	ns
	latency	ns	(0.03)	ns
IC7: lateral visual	height	ns	0.001*	ns
	latency	ns	ns	ns

ns = not significant; parentheses = trending; *p < 0.005; **p < 0.0005
